# Supplementary material for: Correlation between neonatal hyperbilirubinemia and vitamin D levels: A meta-analysis
Source: PLoS One. 2021 May 27;16(5):e0251584. doi: 10.1371/journal.pone.0251584 (PMC8158937; doi:10.1371/journal.pone.0251584)
Supplement: S1 File — (ZIP) [file pone.0251584.s002.zip › search method.docx]

| # 8 | **[31](http://apps.webofknowledge.com/summary.do?product=UA&doc=1&qid=3778&SID=7EvriLDvKxrFtoKOXzn&search_mode=CombineSearches&update_back2search_link_param=yes)** | #7 AND #6 AND #5  *数据库= WOS, DIIDW, KJD, MEDLINE, RSCI, SCIELO 时间跨度=所有年份*  *检索语言=自动* |  |  |
| --- | --- | --- | --- | --- |
|  | | | | |
| # 7 | **[182,911](http://apps.webofknowledge.com/summary.do?product=UA&doc=1&qid=3777&SID=7EvriLDvKxrFtoKOXzn&search_mode=AdvancedSearch&update_back2search_link_param=yes)** | TS=(Vitamin D)  *数据库= WOS, DIIDW, KJD, MEDLINE, RSCI, SCIELO 时间跨度=所有年份*  *检索语言=自动* |  |  |
|  | | | | |
| # 6 | **[913,031](http://apps.webofknowledge.com/summary.do?product=UA&doc=1&qid=3775&SID=7EvriLDvKxrFtoKOXzn&search_mode=AdvancedSearch&update_back2search_link_param=yes)** | TS=(Infant, Newborn OR Infants, Newborn OR Newborn Infant OR Newborn Infants OR Newborns OR Neonate OR Neonates OR Newborn)  *数据库= WOS, DIIDW, KJD, MEDLINE, RSCI, SCIELO 时间跨度=所有年份*  *检索语言=自动* |  |  |
|  | | | | |
| # 5 | **[17,130](http://apps.webofknowledge.com/summary.do?product=UA&doc=1&qid=3760&SID=7EvriLDvKxrFtoKOXzn&search_mode=AdvancedSearch&update_back2search_link_param=yes)** | TS=(Hyperbilirubinemia OR Hyperbilirubinemias OR Bilirubinemia OR Bilirubinemias)  *数据库= WOS, DIIDW, KJD, MEDLINE, RSCI, SCIELO 时间跨度=所有年份* |  |  |

**Search method**

**Web of science**
