# Supplementary material for: Correlation between neonatal hyperbilirubinemia and vitamin D levels: A meta-analysis
Source: PLoS One. 2021 May 27;16(5):e0251584. doi: 10.1371/journal.pone.0251584 (PMC8158937; doi:10.1371/journal.pone.0251584)
Supplement: Attachment — Submitted filename: renamed_64905.docx [file pone.0251584.s003.docx]

Dear Editors and Reviewers:

 Thank you for your letter and for the Editors and reviewers’ comments concerning our manuscript entitled ”Correlation between neonatal hyperbilirubinemia and vitamin D levels: A meta-analysis”(ID: PONE-D-20-33494). Those comments are all valuable and very helpful for revising and improving our paper, as well as the important guiding significance to our researches. We have studied comments carefully and have made correction which we hope meet with approval. Revised portion are marked in red in the paper. The main corrections in the paper and the responds to the reviewer’s comments are as flowing:

Responds to the editor’s comments:

1. Response to comment 1: Please ensure that your manuscript meets PLOS ONE's style requirements, including those for file naming. Response: It is really true as Editor’s suggest, We have re-read the article, and the style of the article and title meets the requirements of Plos one.
2. Response to comment 2: At this time, we ask that you please provide the full search strategy and search terms for at least one database used as Supplementary Information.. Response: We accept Editor’s good suggestion, we submitted the search method of Web of science in the supplementary file.
3. Response to comment 3: Please confirm that you have included all items recommended in the PRISMA checklist including details of reasons for study exclusions in the PRISMA flowchart and number of studies excluded for each reason. Response: Editor’s suggestion is very good, we checked it carefully,we are sure we have included all items recommended in the PRISMA checklist including details of reasons for study exclusions in the PRISMA flowchart and number of studies excluded for each reason.
4. Response to comment 4: Please include captions for your Supporting Information files at the end of your manuscript, and update any in-text citations to match accordingly. Please see our Supporting Information guidelines for more information.

Response: We accept Editor’s suggestion, we have added the title in the supplementary file to the end of the article.

Responds to the Reviewer’s comments:

1. Reviewer's Responses to Questions

Comments to the Author

1. Is the manuscript technically sound, and do the data support the conclusions?

The manuscript must describe a technically sound piece of scientific research with data that supports the conclusions. Experiments must have been conducted rigorously, with appropriate controls, replication, and sample sizes. The conclusions must be drawn appropriately based on the data presented.

Response: The data of the manuscript is done strictly in accordance with the meta-analysis process, and the data can support its conclusion.

2. Has the statistical analysis been performed appropriately and rigorously?

Response: The meta-analysis strictly follows the process for statistical analysis.

3. Have the authors made all data underlying the findings in their manuscript fully available?

Response: Thank you for your affirmation of my rigorous work.

4. Is the manuscript presented in an intelligible fashion and written in standard English?

Response: The manuscript has been polished by professional English personnel, and its language is in line with standard English.

PLOS ONE does not copyedit accepted manuscripts, so the language in submitted articles must be clear, correct, and unambiguous. Any typographical or grammatical errors should be corrected at revision, so please note any specific errors here.

Reviewer #1:

 1.  Response to comment 1: Suggestions on Abstract: The abstract submitted does not have a purpose or background. Suggested to include few lines on neonatal hyperbilirubinemia and why it is important to identify if there is any correlation between the two. Response:It is really true as Reviewer suggested that we should modify the introduction section. We have re-written this part according to the Reviewer’s suggestion, refer the red letter section on line37-39 of page 4 of the article for details.

2. Response to comment 2: For the results stated in the abstract and in the main paper, would recommend to include the SI units for the vitamin d levels. Were the levels of Vitamin D reported in all the papers with the same metric measurements? Response: We are very sorry for our negligence of this problem. We have added the quotation, refer the red letter section on line 47, line53, line185, line206, line209, line232 and line234.
3. Response to comment 3: Suggestions to modify the conclusion for the abstract: Vitamin D levels were observed to be lower in neonates with hyperbilirubinemia as compared to term neonates without hyperbilirubinemia in this study. This can possibly suggest that neonates with lower vitamin D levels are at higher risk for developing hyperbilirubinemia Response: It is really true as Reviewer suggestion, We have made correction according to the Reviewer’s comments. refer Line 54-56 for details
4. Response to comment 4: I4) Corrections: Line 42: A meta-analysis was conducted on the included studies using Stata11.0 43 software. Line 48: Vitamin D level of infants with hyperbilirubinemia (15 to 20 mg/dl group?) was 9.52 (Z =15.55, 95% CI-10.72~-8.32, P<0.05) lower than that of healthy infants. Line 50: It is unclear what these results are? vitamin D level of hyperbilirubinemia neonates were 5.35 lower than that of healthy neonates (Z =6.43, 95% CI-6.98~-3.72, 51 P<0.05). Response: It is really true as Reviewer advices, We have made correction according to the Reviewer’s comments. refer Line44,Line 49-50, Line54-56 for details.
5. Response to comment 5: Introduction: Introduction can be more precise on neonatal hyperbilirubinemia and vitamin D levels. More on why the correlation needs to be done. The introduction of the manuscript is very elaborate. Response: Considering the Reviewer’s suggestion, We need to explain. We have a description in the article,refer Line96-98 for details.
6. Response to comment 6: When the number of studies included in the analysis is less than 10, the Hartung-Knapp-Sidik-Jonkman method can better reduce the risk of type 1 error than the DerSimonian and Laird method. What method did the authors use for the random effect? Response: We use the D-L method, which belongs to the random effects model. In the weight distribution, the small sample research obtains more weight than the fixed effects model, while the large sample research relatively reduces the weight, so that the weights obtained between the studies, regardless of the sample size, the difference is smaller than the fixed effect model, so the interpretation of the results should be more conservative and cautious
7. Response to comment7: Funnel plot is recommended only when more than 10 articles are included in the meta-analysis. Can authors explain why they chose to subgroup and do a funnel plot for only two studies and four studies? Response: Above the Reviewer’s suggestion, We need to explain. We not only use the funnel chart to analyze the publication bias in the article, but also use the Egger method to test it.

Special thanks to you for your good comments.

Reviewer #2:

Response to comment: The main concern about this article is it does not describe the units properly. For eg, it states: The vitamin D level of infants with hyperbilirubinemia was 9.52 (Z =15.55, 95% CI-10.72~-8.32, P<0.05) lower than that of healthy infants. What is 9.52? is it 9.52 times, is the vitamin D level 9.52, if so in what units? The way it is written it is very hard to understand. Also of the articles selected for the metanalysis, no description is given of how they measured vitamin D levels. Generally, there is considerable variation in the assays used with mass spec being the most accurate. Response: We are very sorry for our negligence of this problem. We have added the quotation, refer the red letter section on line 47, line53, line185, line206, line209, line232 and line234.

Thank you very much for your careful reading and your valuable comments to us.

We tried our best to improve the manuscript and made some changes in the manuscript. These changes will not influence the content and framework of the paper. We appreciate for Editors and Reviewers’warm work earnestly, and hope that the correction will meet with approval.

Once again, thank you very much for your comments and suggestions.

Yours sincerely,

Changjun Ren

Corresponding author:

Name: Changjun Ren

E-mail: 137544907@qq.com
